# Supplementary material for: Shared and distinct genetic etiologies for different types of clonal hematopoiesis
Source: Nat Commun. 2023 Sep 8;14:5536. doi: 10.1038/s41467-023-41315-5 (PMC10491829; doi:10.1038/s41467-023-41315-5)
Supplement: Supplementary file 3 — Description of Additional Supplementary Files [file 41467_2023_41315_MOESM3_ESM.pdf]

Description of Additional Supplementary Files

File Name: Supplementary Data 1
Description: Pairwise genetic correlations between each type of clonal hematopoiesis, telomere length, and 19 blood cell traits. The upper diagonal gives the genetic correlation, and the lower diagonal gives the -log10(P) value derived using the high-definition likelihood (HDL) method. The genetic correlation display color reflects the magnitude of association.

File Name: Supplementary Data 2
Description: Pairwise genetic correlations between each type of clonal hematopoiesis, telomere length, and 19 blood cell traits. The upper diagonal gives the genetic correlation, and the lower diagonal gives the -log10(P) value derived using linkage disequilibrium score regression (LDSC). The genetic correlation display color reflects the magnitude of association.

File Name: Supplementary Data 3
Description: Overlapping counts of each CH type in UK Biobank.

File Name: Supplementary Data 4
Description: Pairwise phenotypic associations between each type of clonal hematopoiesis, telomere length, and 19 blood cell traits. The upper diagonal gives the T-statistic, and the lower diagonal gives the -log10(P) value derived using linear regression adjusted for age, age-squared, 25-level smoking status, and sex (in non LOY or LOX comparisons). The T-statistic display color reflects the magnitude of association.

File Name: Supplementary Data 5
Description: Linear associations between LOY and CHIP by CHIP gene. Associations were adjusted for age, age-squared, and detailed smoking status and restricted to males.

File Name: Supplementary Data 6
Description: Exome-wide rare non-synonymous gene burden tests of LOY. All models were adjusted for age, age-squared, WES batch, and the first ten genetic ancestry principal components (PCs) as generated by Bycroft et al.

File Name: Supplementary Data 7
Description: Association between protein truncating variant (PTV) carrier status and PAR-LOY overall and across 6 additional criteria. All association tests were run separately for each gene: ASXL1, DNMT3A, TET2, and GIGYF1.

File Name: Supplementary Data 8
Description: Proportion of individuals with each type of CH by autosomal mCA cell fraction (CF) categories.

File Name: Supplementary Data 9
Description: Proportion of individuals with each type of CH by CHIP variant allele fraction (VAF) categories.

File Name: Supplementary Data 10
Description: Frequency of CHIP gene mutations by autosomal mCA status.

File Name: Supplementary Data 11
Description: Frequency of autosomal mCA mutations by CHIP status.

File Name: Supplementary Data 12
Description: CHIP VAF association (β) with 95% confidence intervals by CHIP and autosomal mCA status. Associations are adjusted for age, age-squared, sex, and detailed smoking status.

File Name: Supplementary Data 13
Description: Incident lymphoid and myeloid malignancy associations (HR) with 95% confidence intervals by CHIP and autosomal mCA status. Associations were derived using Cox proportional hazards regression adjusted for age, age-squared, 25-level smoking status, and sex.

File Name: Supplementary Data 14
Description: Pathways and gene sets significantly associated with LOY.

File Name: Supplementary Data 15
Description: Pathways and gene sets significantly associated with LOX.

File Name: Supplementary Data 16
Description: Pathways and gene sets significantly associated with autosomal mCAs.

File Name: Supplementary Data 17
Description: Pathways and gene sets significantly associated with CHIP.

File Name: Supplementary Data 18
Description: Pathways and gene sets significantly associated with MPN.

File Name: Supplementary Data 19
Description: Mendelian randomization results between telomere length and each type of CH using telomere length instrumental SNPs with Steiger and Radial filters.

File Name: Supplementary Data 20
Description: Average telomere length by CHIP VAF categories.

File Name: Supplementary Data 21
Description: Average telomere length by autosomal mCA CF categories.

File Name: Supplementary Data 22
Description: Telomere length association (β) with 95% confidence intervals by CHIP and autosomal mCA status. Associations are adjusted for age, age-squared, sex, and detailed smoking status.

File Name: Supplementary Data 23
Description: Multi-trait analysis of GWAS (MTAG) results for MPN using LOY and TL summary statistics.

File Name: Supplementary Data 24
Description: Bayesian test for colocalization between LOY and MPN using the coloc R package.

File Name: Supplementary Data 25
Description: Bayesian test for colocalization between TL and MPN using the coloc R package.

File Name: Supplementary Data 26
Description: Hematopoietic phenotype GWAS population and sample size.

File Name: Supplementary Data 27
Description: Gene list utilized for CHIP calling within 198,178 UK Biobank participants. 7,280 (3.7%) individuals were found to have at least one CHIP curated variant. The number of individuals detected with each gene mutation are provided.

File Name: Supplementary Data 28
Description: Driver gene mutations detected within 198,178 UK Biobank participants.

File Name: Supplementary Data 29
Description: Observed heritability for each type of CH, telomere length, and 19 blood cell traits derived using the high-definition likelihood (HDL) method.

File Name: Supplementary Data 30
Description: Pairwise hematopoietic phenotype correlations and intercept values used to constrain the high-definition likelihood (HDL) and LD score regression (LDSC) intercepts. The sample size for each phenotype is the number of participants from each GWAS study. Pearson correlations were calculated between phenotypes using 482,378 UK Biobank participants.
