# Supplementary material for: Shared and distinct genetic etiologies for different types of clonal hematopoiesis
Source: Nat Commun. 2023 Sep 8;14:5536. doi: 10.1038/s41467-023-41315-5 (PMC10491829; doi:10.1038/s41467-023-41315-5)
Supplement: Supplementary file 5 — Reporting Summary [file 41467_2023_41315_MOESM5_ESM.pdf]

nature portfolio

Corresponding author(s): John R. B. Perry, Vijay G. Sankaran, Mitchell J. Machiela

Last updated by author(s): Aug 11, 2023

Reporting Summary

Nature Portfolio wishes to improve the reproducibility of the work that we publish. This form provides structure for consistency and transparency in reporting. For further information on Nature Portfolio policies, see our Editorial Policies and the Editorial Policy Checklist.

Statistics

For all statistical analyses, confirm that the following items are present in the figure legend, table legend, main text, or Methods section.

n/a	Confirmed	
☐	☒	The exact sample size (*n*) for each experimental group/condition, given as a discrete number and unit of measurement
☒	☐	A statement on whether measurements were taken from distinct samples or whether the same sample was measured repeatedly
☐	☒	The statistical test(s) used AND whether they are one- or two-sided<br>*Only common tests should be described solely by name; describe more complex techniques in the Methods section.*
☐	☒	A description of all covariates tested
☐	☒	A description of any assumptions or corrections, such as tests of normality and adjustment for multiple comparisons
☐	☒	A full description of the statistical parameters including central tendency (e.g. means) or other basic estimates (e.g. regression coefficient) AND variation (e.g. standard deviation) or associated estimates of uncertainty (e.g. confidence intervals)
☐	☒	For null hypothesis testing, the test statistic (e.g. *F*, *t*, *r*) with confidence intervals, effect sizes, degrees of freedom and *P* value noted<br>*Give P values as exact values whenever suitable.*
☒	☐	For Bayesian analysis, information on the choice of priors and Markov chain Monte Carlo settings
☒	☐	For hierarchical and complex designs, identification of the appropriate level for tests and full reporting of outcomes
☒	☐	Estimates of effect sizes (e.g. Cohen's *d*, Pearson's *r*), indicating how they were calculated

Our web collection on statistics for biologists contains articles on many of the points above.

Software and code

Policy information about availability of computer code

Data collection	N/A - no software was used to collect data for this study.
Data analysis	LDSC(v1.0.1), BOLT-LMM(v2.3.6), Mutect2, R(3.6.3), Python(2.7.18), GCTA(1.92.0 beta2), TwoSampleMR(v0.5.6), sARTP(v2), HDL(v1.0.1)

For manuscripts utilizing custom algorithms or software that are central to the research but not yet described in published literature, software must be made available to editors and reviewers. We strongly encourage code deposition in a community repository (e.g. GitHub). See the Nature Portfolio guidelines for submitting code & software for further information.

Data

Policy information about availability of data

All manuscripts must include a data availability statement. This statement should provide the following information, where applicable:

- Accession codes, unique identifiers, or web links for publicly available datasets
- A description of any restrictions on data availability
- For clinical datasets or third party data, please ensure that the statement adheres to our policy

All data used in the analysis is available from UK Biobank upon request (https://www.ukbiobank.ac.uk)

Research involving human participants, their data, or biological material

Policy information about studies with human participants or human data. See also policy information about sex, gender (identity/presentation), and sexual orientation and race, ethnicity and racism.

Reporting on sex and gender	Sex was used to report findings based on biologic attribute. Any sex-specific analyses were clearly annotated in the manuscript.
Reporting on race, ethnicity, or other socially relevant groupings	We reported on individuals of European ancestry, defined as individuals carrying alleles at ancestry informative markers that cluster near known reference European population samples. We restricted analyses to individuals of European ancestry to avoid confounding due to ancestry.
Population characteristics	UK Biobank is a national resource that has been described elsewhere (https://www.ukbiobank.ac.uk). Individuals were not directly selected for inclusion in the study on the basis of any disease or health parameter.
Recruitment	UK Biobank recruited all individuals aged 40-69 years who were registered with the National Health Service and living up to ~25 miles from on of the 22 study assessment centers were invited to participate in 2006-2010. Overall, about 9.2 million invitations were mailed to recruit 503,325 participants (a response rate of 5.47%).
Ethics oversight	UK Biobank has approval from the Northwest Multi-centre Research Ethics Committee (MREC) as a Research Tissue Bank (RTB) approval. This approval means that researchers do not require separate ethical clearance and can operate under the RTB approval. See https://www.ukbiobank.ac.uk/learn-more-about-uk-biobank/about-us/ethics for more details.

Note that full information on the approval of the study protocol must also be provided in the manuscript.

Field-specific reporting

Please select the one below that is the best fit for your research. If you are not sure, read the appropriate sections before making your selection.

☒ Life sciences ☐ Behavioural & social sciences ☐ Ecological, evolutionary & environmental sciences

For a reference copy of the document with all sections, see nature.com/documents/nr-reporting-summary-flat.pdf

Life sciences study design

All studies must disclose on these points even when the disclosure is negative.

Sample size	We used the full available sample in the UK Biobank (N=482,378) for phenotypic analyses after removing individuals with sex discordance or whose DNA failed genotyping quality control.
Data exclusions	Only individuals failing standard genotyping quality control parameters defined initially by the UK Biobank study were excluded from analysis.
Replication	We did not perform replication analyses for our study as well-powered samples were unavailable.
Randomization	N/A as the study is based on a prospective cohort.
Blinding	All analysts participating in calling procedures were blinded to other clinical and patient features of analytic interest.

Reporting for specific materials, systems and methods

We require information from authors about some types of materials, experimental systems and methods used in many studies. Here, indicate whether each material, system or method listed is relevant to your study. If you are not sure if a list item applies to your research, read the appropriate section before selecting a response.

Materials & experimental systems

n/a	Involved in the study	
☒	☐	Antibodies
☒	☐	Eukaryotic cell lines
☒	☐	Palaeontology and archaeology
☒	☐	Animals and other organisms
☒	☐	Clinical data
☒	☐	Dual use research of concern
☒	☐	Plants

Methods

n/a	Involved in the study	
☒	☐	ChIP-seq
☒	☐	Flow cytometry
☒	☐	MRI-based neuroimaging
